# Supplementary material for: Health and economic burden of canine rabies in Chilga district, centeral Gondar Zone, Amhara Region, Ethiopia
Source: PLoS Negl Trop Dis. 2026 Apr 20;20(4):e0014196. doi: 10.1371/journal.pntd.0014196 (PMC13124050; doi:10.1371/journal.pntd.0014196)
Supplement: S1 File — (DOCX) [file pntd.0014196.s001.docx]

# 7. APPENDICES

Appendix 1: Questionnaire to be administered to households about to rabies victims/families

Questions to rabies victims/their families exposed between September 2017 and September 2019

*Respondent to participate:  Agree* ---------Disagree--------- *Date of interview* ---------------------- Name of respondent (household head) -----------------------------------------district--------------------kebele--------------Sex-----------age---------address/phone No---------------------------------

**Human (Part one)**

1. Do you know, what rabid dog/animal means? A. Yes B. No

If yes, what are typical symptoms: 1. A dog disease with aggressiveness and salivation

2. A dog disease with paralysis and salivation

3. A dog disease with restlessness and salivation

2. Were/was there any family member bitten/suspected to be bitten by rabid dog since September 2017? A. Yes B. No

For each bitten or suspected of bitten (exposed) family member complete the following table

| No. | Status of  exposure (as non bleeding, bleeding scratch, open wound) | Status of the dog(confirmed, only clinically suspected, later found non rabid | Date of  exposure (at least month) | Age of the victim | Sex of the victim | Site of exposure  such as   (Head…) | Measure taken during exposure (wound management and or PEP and or traditional treatment ) | Fate of the victim |
| --- | --- | --- | --- | --- | --- | --- | --- | --- |
|  |  |  |  |  |  |  |  |  |
|  |  |  |  |  |  |  |  |  |
|  |  |  |  |  |  |  |  |  |
|  |  |  |  |  |  |  |  |  |

For each exposed family number complete the following cost related table

| Victim no | Convention treatment | | | | | | Traditional treatment | | | |
| --- | --- | --- | --- | --- | --- | --- | --- | --- | --- | --- |
|  | Wound care cost if any | No of PEP doses if any | Hospital cost (drug and bed) cost of any | No of days of illness | The expense of the accompany during period of illness | Transport cost of the victim and his accompany | Diagnosis, Treatment cost | Transport cost | Days of off home treatment | Expense of the accompany |
|  |  |  |  |  |  |  |  |  |  |  |
|  |  |  |  |  |  |  |  |  |  |  |
|  |  |  |  |  |  |  |  |  |  |  |
|  |  |  |  |  |  |  |  |  |  |  |
|  |  |  |  |  |  |  |  |  |  |  |

**Livestock (part two)**

3. Were/was there any rabid/rabies suspected cases among your animal since September 2017?

A. Yes B. No

If yes, complete the following table

| No | Date of exposure(atleast month) | Species of the  animal | Age of animal | Sex of animal | Status of the animal(rabid or suspected) | The nature of exposure (minor, Moderate, severe) | If treated conventionally cost of treatment | If treated traditionally cost of treatment | No of days spent for get the affected animal treated | If died the estimated price | If Salvaged the estimate value lost |
| --- | --- | --- | --- | --- | --- | --- | --- | --- | --- | --- | --- |
|  |  |  |  |  |  |  |  |  |  |  |  |
|  |  |  |  |  |  |  |  |  |  |  |  |
|  |  |  |  |  |  |  |  |  |  |  |  |

**Questionnaire to be administered to households about traditional practice**

1. Do you know traditional healers who give anti-rabies traditional medicine?

A. Yes B. No

2. Mostly, what do you and your community do when humans/animals are exposed to rabies?

A. traditional treatment B. modern treatment in health centers

2.1. If traditional does it include diagnosis or only treatment?

A. only treatment B. both treatment and diagnosis

2.2. If traditional, what type of treatment is used in the traditional method?

A. spiritual B. non spiritual C. mixed

2.3 If the treatment is non spiritual, what is the medicine made of:

A. herbs B. animal products C. others

3. Have you/families diagnosed/treated when suspected/bitten by rabid dog?

A.Yes B. No

3.1. If yes, do you have experience of misdiagnosis in the traditional treatment?

A. Yes B. No

  3.2. If yes, do you have experience of treatment failure in the traditional treatment?

A. Yes B. No

If yes, what do you think is the failure?

------------------------------------------------------------------------------------------------------------------

3.3. Do you have experience of success in traditional treatment? A. Yes B. No

4. Do you have experience of failure after modern treatment of human rabies exposure cases?

A. Yes B. No

If you yes, what do you think will be the reason------------------------------------------------------------------------------------------------------------------------------------------------------------------------

5. Do you have experience of failure after modern treatment of animal rabies exposure cases?

B. Yes B. No

If you yes, what do you think will be the reason-------------------------------------------------------------------------------------------------------------------------------------------------------------------

6. Do you have experience of success after modern treatment human exposure cases?

A. yes B. No

7. Do you have experience of success after modern treatment animal exposure cases?

A. Yes B. No

8. Do you think that traditional healers can cure human bitten by a rabid animal?

A. Yes B. No

9. Do you think that traditional healers can cure an animal bitten by a rabid animal?

A. Yes B. No

10. Do you think that traditional healers can cure human once started to show clinical signs of the rabies? A. Yes B. No

11. Do you think that traditional healers can cure an animal once started to show clinical signs of the rabies? A. Yes B. No

12. If using traditional treatment in humans does it have side effect in humans? A. Yes B. No

If yes what ?----------------------------------------------------------------------------------------------------------------------------------------------------------------------------------------------------------------

13. If using traditional treatment in animals does it have side effect? A. Yes B. No

14. Have ever used preventive traditional medicine for rabies i.e. medicine taken before any suspected exposure of rabies? A. Yes B. No

15. If yes do you think it is effective. A. Yes B. No

In traditional treatment how do traditional healers determine the dose? ------------------------------------------------------------------------------------------------------------------------

16. How medicine administration performed? A. by healers B. by patients/them selves

17. Would they use anti-dotes? A. Yes B. No

18. If yes, what are? ----------------------------------------------------------------------------
