# Supplementary material for: Health and economic burden of canine rabies in Chilga district, centeral Gondar Zone, Amhara Region, Ethiopia
Source: PLoS Negl Trop Dis. 2026 Apr 20;20(4):e0014196. doi: 10.1371/journal.pntd.0014196 (PMC13124050; doi:10.1371/journal.pntd.0014196)
Supplement: Attachment — Submitted filename: PNTD-D-25-01493_PEER reviewer.pdf [file pntd.0014196.s003.pdf]

PLOS Neglected Tropical Diseases

Health and Economic Burden of Canine Rabies in Chilga District, Centeral Gondar Zone, Amhara Region, Ethiopia

--Manuscript Draft--

**Manuscript Number:**	PNTD-D-25-01493
**Full Title:**	Health and Economic Burden of Canine Rabies in Chilga District, Centeral Gondar Zone, Amhara Region, Ethiopia
**Short Title:**	Health and Economic Burden of Canine Rabies
**Article Type:**	Research Article
**Keywords:**	Disability Adjusted Life Years; Economic burden; Public health burden; Livestock; Lyssavirus; Rabies
**Abstract:**	Rabies is a deadly neglected tropical disease caused by a Lyssavirus that affects all warm-blooded animals, including humans. While canine rabies is prevalent in Ethiopia, its public health and economic burden is not well understood due to a lack of reliable surveillance data. This study aimed to estimate the economic and public health burden of canine rabies in Chilga district, Northwest Ethiopia. The study was conducted retrospectively over two years, from September 2017 to September 2019. A multistage cluster sampling approach was used to select representative households for data collection. A total of 768 households' heads were interviewed to collect data about rabies in their family members, dogs and livestock. The public health burden of the disease was estimated in disability adjusted life years (DALYs), while the economic burden was estimated by aggregating the direct and indirect cost of rabies in humans, livestock, and dogs determined using cost estimation models. The annual human rabies death in the district was estimated at 37deaths/100,000 populations per year. The overall public health burden of canine rabies was 1576.5 DALYs/100,000 people per year. The economic burden estimates at household and district level were 457.4 Ethiopian birr (USD 16.3) and 14.9million 4 Ethiopian birr (USD 0.53million) per year respectively. The largest portion (76.7%) of the economic burden was contributed by livestock losses, while the smallest portion (~0.3%) was by the cost of dog's vaccination. Generally, the study revealed a significant public health and economic burden estimates of canine rabies in the district that needs a control intervention focus on dog vaccination, which was observed to be very low, could have significant impact in reducing both public health and economic burden.
**Additional Information:**	
**Question**	**Response**
**Government Employee**<br><br>Are you or any of the contributing authors an employee of the United States government?<br><br>Manuscripts authored by one or more US Government employees are not copyrighted, but are licensed under a CC0 Public Domain Dedication, which allows unlimited distribution and reuse of the article for any lawful purpose. This is a legal requirement for US Government employees.	No - No authors are employees of the U.S. government.

Powered by Editorial Manager® and ProduXion Manager® from Aries Systems Corporation

This will be typeset if the manuscript is accepted for publication.	
**Financial Disclosure**<br><br>Enter a financial disclosure statement that describes the sources of funding for the work included in this submission. Review the submission guidelines for detailed requirements. View published research articles from *PLOS Neglected Tropical Diseases* for specific examples.<br><br>This statement is required for submission and **will appear in the published article** if the submission is accepted. Please make sure it is accurate.<br><br>**Funded studies**<br>Enter a statement with the following details:<br>• Initials of the authors who received each award<br>• Grant numbers awarded to each author<br>• The full name of each funder<br>• URL of each funder website<br>• Did the sponsors or funders play any role in the study design, data collection and analysis, decision to publish, or preparation of the manuscript?<br><br>Did you receive funding for this work?	The author(s) received no specific funding for this work.
**Competing Interests**<br><br>On behalf of all authors, disclose any competing interests that could be perceived to bias this work.<br><br>This statement will be typeset if the manuscript is accepted for publication.<br><br>Review the instructions link below and PLOS NTDs' competing interests policy to determine what information must be disclosed at submission.	The authors have declared that no competing interests exist.
**Data Availability**	All relevant data within the manuscript and its Supporting Information files, which are necessary to replicate the findings of this study, will be publicly available without

Provide a **Data Availability Statement** in the box below. This statement should detail where the data used in this submission can be accessed. This statement will be typeset if the manuscript is accepted for publication.<br><br>Before publication, authors are required to make all data underlying their findings fully available, without restriction. Review our PLOS Data Policy page for detailed information on this policy. Instructions for writing your Data Availability statement can be accessed via the Instructions link below.	restriction at the time of publication.

Powered by Editorial Manager® and ProduXion Manager® from Aries Systems Corporation

Manuscript
Click here to access/download;Manuscript;Canine Rabies Manuscript.pdf

Health and Economic Burden of Canine Rabies in Chilga District, Centeral Gondar Zone, Amhara Region, Ethiopia

Tewodros Birhan Engida[1, 2], Amsalu Misgie Molla[3] * , Wassie Molla[2], Wudu Temesgen Jemberu[2,4]

1. Chilga Animal Resources development and Promotion Office, Chilga Woreda, Amhra Region, Central Gondar Zone, Ethiopia
2. Department of Veterinary Epidemiology and Public Health, College of Veterinary Medicine and Animals Sciences, University of Gondar, P. O. Box 196, Gondar, Ethiopia
3. Department of Veterinary Science, College of Agriculture and Environmental Sciences, Debre Tabor University, P. O. Box 272, Debre Tabor, Ethiopia.
4. International Livestock Research Institute, P.O. Box 5689, Addis Ababa, Ethiopia.

*** corresponding author: Amsalu Misgie Molla; email: amsaluvet21@gmail.com**

ABSTRACT

Rabies is a deadly neglected tropical disease caused by a Lyssavirus that affects all warm-blooded animals, including humans. While canine rabies is prevalent in Ethiopia, its public health and economic burden is not well understood due to a lack of reliable surveillance data. This study aimed to estimate the economic and public health burden of canine rabies in Chilga district, Northwest Ethiopia. The study was conducted retrospectively over two years, from September 2017 to September 2019. A multistage cluster sampling approach was used to select representative households for data collection. A total of 768 households' heads were interviewed to collect data about rabies in their family members, dogs and livestock. The public health burden of the disease was estimated in disability adjusted life years (DALYs), while the economic burden was estimated by aggregating the direct and indirect cost of rabies in humans, livestock, and dogs determined using cost estimation models. The annual human rabies death in the district was estimated at 37deaths/100,000 populations per year. The overall public health burden of canine rabies was 1576.5 DALYs/100,000 people per year. The economic burden estimates at household and district level were 457.4 Ethiopian birr (USD 16.3) and 14.9million 4 Ethiopian birr (USD 0.53million) per year respectively. The largest portion (76.7%) of the economic burden was contributed by livestock losses, while the smallest portion (~0.3%) was by the cost of dog's vaccination. Generally, the study revealed a significant public health and economic burden estimates of canine rabies in the district that needs a control intervention focus on dog vaccination, which was observed to be very low, could have significant impact in reducing both public health and economic burden.

Key words: *DALY, Economic burden, Public health burden, Livestock, Lyssavirus, Rabies*

AUTHOR SUMMARY

Rabies, caused by the Lyssavirus, affects all warm-blooded animals, including humans, and poses a significant public health threat in Ethiopia, particularly due to canine rabies. This study assessed the economic and health burdens of canine rabies using a multistage cluster sampling method to interview 768 household heads. The findings revealed a public health burden of 37 rabies deaths per 100,000 people annually, resulting in 1,576.5 Disability Adjusted Life Years (DALYs) lost per 100,000. The economic burden was estimated at 457.4 Ethiopian birr (USD 16.3) per household and 14.9 million birr (USD 0.53 million) for the district, primarily due to livestock losses, which accounted for 76.7%. The study highlights the urgent need for interventions, particularly dog vaccination, to mitigate the public health and economic burdens of rabies.

1. INTRODUCTION

Rabies is a neglected tropical disease caused by a member of the *Lyssavirus* genus in the *Rhabdo viridae* family [1, 2]. It is the most serious zoonotic disease that someone might encounter [3]. Naturally, the virus circulates among animals such as dogs, cats, ferrets, fox, raccoon, mongooses, bats, and wolves [4, 5], but it can be spilled over to humans through these animals, particularly those of dogs [6]. The animal and human usually contract rabies from the bite of an infected animal. The virus may enter into the body if the mucous membranes or a scratch or breaks in the skin have contact with saliva containing the rabies virus [7]. The most frequent route by which the virus transmit to human is through an animal bite [6]. A human infection causes fatal encephalomyelitis [8], which is expressed by confusion, aggressive behavior, and extremely painful laryngeal spasms [6, 9]. Once clinical signs appear, fatality rate is almost 100% [10].

Estimations on the global burden of rabies indicate a health impact of 59,000 human deaths per year, a loss of 3.7 million DALYs per year and economic loss of about 8.6 billion USD per year mainly due to premature death and post-exposure treatment costs [11]. Most of the human death and economic loss occur in Asian and African countries [12, 13].

Canine rabies is an economically unique zoonosis because most of its associated costs do not result from illness, but are the consequence of human deaths and efforts to prevent the disease in humans, livestock and companion animals. This unique pattern of costs reflects two basic facts: the case

fatality rate of rabies is nearly 100%, and the disease is completely preventable through timely post exposure prophylaxis (PEP) with rabies vaccine [14]. As a result, many individuals who are at very low risk of developing the disease still seek post exposure vaccination, regardless of the recommendation of health professionals [15].

At present, the burden of the disease is high to people who least afford vaccination. Improving the availability of PEP could reduce the number of human deaths, but it is costly. The incidence of dog-mediated rabies can be reduced by sustained mass dog vaccination, and the cost of PEP will would decrease with time if the vaccination is done appropriately [16]. National dog vaccination programs and better access to PEP require consistent and sustained commitment but will have widespread health benefits, particularly for the poorest communities in the world [16].

Ethiopia is one of the worst rabies affected country [11], with domestic dogs being the major sources of infection to humans. Dog management is poor and anti-rabies dog vaccination is generally limited to a small number of dogs found in urban settings [17]. The large dog population size in combination with poor dog management contributes to a high endemicity of canine rabies in Ethiopia [18].In Ethiopia, the national annual estimates from official reports indicate 12 exposure cases and 1.6 rabies deaths per 100,000 populations [19]. However, the actual numbers are expected to be higher as many cases are not reported [20].

The reliability of the reported incidence data is expected to differ among the regions in Ethiopia due to geographical as well as cultural differences. For instance, in rural area, individuals who are exposed to rabies often prefer to see traditional healers for the diagnosis and treatment of the disease because of cultural background, lack of knowledge, limited accessibility to conventional medical treatment. These widespread traditional practices of handling rabies cases might interfere with medical treatment, resulting in an underreporting of the actual number of rabies cases and its related health burden [21].

This study was aimed at estimating the public health and economic burden of canine rabies in Chiliga district, central Gondar Zone, northwest Ethiopia.

2. MATERIALS AND METHODS

2.1. Study Area

The study was conducted in Chilga district, central Gondar administrative zone of Amhara region in Ethiopia (Figure 1). The altitude of the study area ranges between 900-2250 m.a.s.l. The district is characterized by mean annual temperature which ranges between 12°C and 29°C and the mean annual rainfall varies from 950 ml to around 1100 ml. Based on the national census conducted by the Central Statistical Agency of Ethiopia [22], the district has a total of 32,496 households with a population of 237,581(120,103 male and female 117,478 female). Livestock play an important role for income-generating activities of the community in the rural area. The total animal population in the district is estimated at 1,195,136 (524,230 cattle, 20,882 equines and 650,024 small ruminants) [23]. There are 49 rural and 6 town kebeles in the district [24].

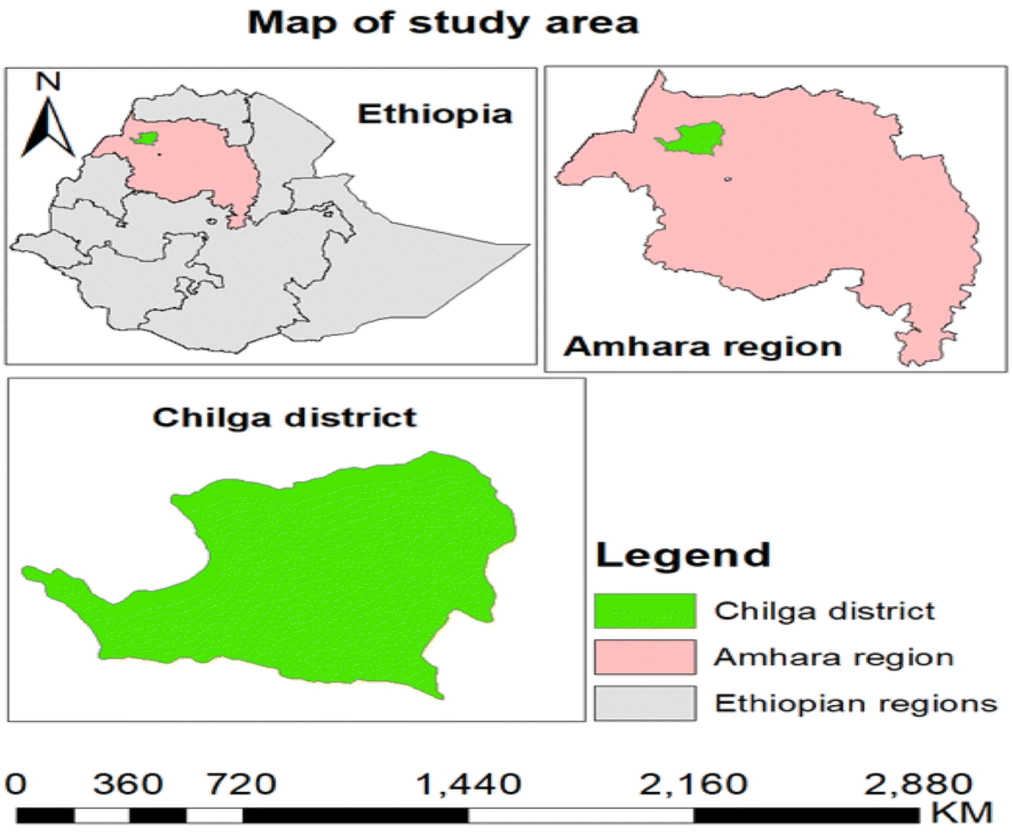

Figure 1: Map of the study district

2.2. Study Design

The study was retrospective study in which rabies related data for the preceding two years (September 2017 to September 2019) were collected through questionnaire survey. Samples of household heads in the study district were interviewed about their experience on the occurrence of rabies and death in their family and livestock to estimate the public health and economic burden of the disease. A cross-sectional questionnaire survey was also undertaken to assess the traditional treatment practices against rabies in the study area.

2.2.1. Sampling techniques and sample size determination

Multistage cluster sampling technique was used to select representative households for data collection. First kebeles were selected by simple random sampling techniques from the district and then from selected kebeles households were selected systematically. Out of 55 ‘Kebeles’ in the district, 25 (23 rural and 2 town) of them were included in the study. From randomly selected kebeles, households were selected systematically. Sample size was determined using Cochran’s sample size formula [25].

$$n = \frac{(t)^2 * P\ (q)}{d^2} \qquad n = \frac{(1.96)^2 0.5(1-0.5)}{(0.05)^2} = 384$$

Where,‘t’ is the value for selected alpha level of 0.025 in each tail = 1.96. (p), (q) is the estimate of variance = 0.25 (assuming 50% of households would experience the disease), d is the acceptable margin of error = 0.05.

The above sample size works for simple random sampling techniques. Nevertheless, the samplin g technique used in the study was multistage cluster sampling and the sample size was adjusted for clustering effect by taking double of the calculated sample size i.e. 768 [26].

2.2.2. The questionnaire survey

The sampled households in the kebele were interviewed using a structured questionnaire (Appendix 1) about their experience of livestock and human rabies in the preceding two years. The questionnaire was developed in English and was translated to the local language, Amharic. The

first question of the questionnaire was posed to ascertain the familiarity of respondent with rabies. That was done by asking the respondent whether he/she is familiar with rabies by using the local name (‘Ye-ebid wusha beshita’) and the main clinical and epidemiological feature of the disease and the response was compared with literature description of rabies clinical signs such as aggressiveness, restlessness, salivation and paralysis. If the answer matched the clinical signs description, the respondent would continue with other part of the questionnaire and if not was excluded from the interview. The remaining part of the questionnaire covered experience of the respondents about rabies exposure, death and treatment/preventive measures (both modern and traditional) in their family members and livestock holding. The questionnaire also included questions related to cost of treatment and livestock deaths or culling (**Appendix 1**).

2.3. Estimating the Public Health Burden of Canine Rabies

Disability-adjusted life years (DALYs) is a method developed by the World Health Organization [27], to calculate the rabies health burden. Disability adjusted life years is the sum of years of life lost (YLL) due to premature death and years lived with disability (YLD) associated with the disease.

$$DALY=YLL + YLD=\sum ((NDeathi * LEi) + (NDisi * D * DW))$$

Where; YLL is calculated as the number of human deaths (Ndeath) within age category (i) multiplied by the life expectancy (LE) of the concerned age category (i), cumulated over all age categories. Eighteen age groups with a 5 years age interval from 0 to 85 years and above were Considered (i = 1 . . . 18) according to the age classification of WHO life expectancy table.

$$YLL=\sum (NDeathi*LEi)$$

Similarly, YLD is calculated by multiplying the total number of disability cases (Ndis) of the concerned age group (*i*), with the duration of the disability (*D* in years) and its corresponding Disability Weight (DW) [12].

2.4. Estimating the Economic Burden of Canine Rabies

The economic burden of the disease associated with both humans and livestock was estimated using the following cost models by adapting from Shwiff *et al*. [28].

Cost of human rabies:

The total cost of human rabies (TC_{RH}) was estimated using model 1.

$$TC_{RH} = NE_m*(DMC+ INMC) +NE_t (CDPH+CTPH+ACPH)…….. (1)$$

Where; NEm=Number of exposed humans that attended modern treatment, DMC=Direct medical cost of rabies exposed human, INMC=Indirect non-medical cost of rabies exposed human, NEt=Number of exposed human that attended traditional treatment, CDPH=Cost of diagnosis per human in traditional treatment, CTPH= Cost of traditional treatment per human, ACPH=Additional costs per human when come to traditional healers (costs of transportation, food).

Cost of dog rabies:

The total cost of rabies in dog (TC_{RD}) in the district was estimated using model 2.

$$TC_{RD} = NDV * (DogV+ VDC + Owner)………………….. (2)$$

Where; NDV=Number of dogs vaccinated in the district, DogV=Cost of dog vaccine, VDC=vaccine delivery cost per dog, Owner=Dog owner cost (travel cost, etc.) per dog.

Cost of rabies in livestock:

The study was considered four livestock species (cattle, equine, goat and sheep), but the disease was reported only in cattle and equines. The total value loss of livestock due to rabies (TC_{RL}) or each livestock species was estimated using model 3.

$$TCRL=\sum_{i=1}^{n}[Price1- Price2]……………………….. (3)$$

Where; n=number of animals exposed, potential price 1 = price before exposure, Price2=culled/salvage value. The cost of livestock price was the sum of cost of rabies in cattle and cost of rabies in equines in Livestock price per head. Price 2 for equines is obviously zero as rabid equines have no salvage value.

The total cost (TC) associated with rabies

The total cost (TC) associated with rabies was estimated by considering the sum of human, dog, and livestock components using model 4.

$$T_C = TC_{RH} + TC_{RD} + TC_{RL} \qquad (4)$$

2.5. Data Management and Analysis

After data collection the data was entered into Microsoft Excel. Then the data was analyzed using descriptive methods. Public health burden was analyzed in terms of DALY/100,000 people per year and economic burden costs were estimated or calculated in terms of Ethiopian Birr (ETB) (the average exchange rate of USD to ETB during the study period was 1USD = 28 ETB) per household and district.

2.6. Ethical Consideration

This work was conducted under the permits of the College of Veterinary Medicine and Animal Sciences Research Ethics Review Committee, reference number 005/2017. During the questionnaire survey, an informed oral consent was obtained from the respondents. Respondents was informed on the purposes of the research, the procedure followed during the interview and on the subsequent use of their responses. It was emphasized that their participation is voluntary and that they could stop with the interview at any time. Permission to conduct the interview was obtained from the district health and livestock development and promotion offices of the district.

3. RESULTS

3.1. Public Health Burden of Rabies

During the study, a total of 768 head of households (respondents) were interviewed about occurrence of rabies in their family and livestock, and anti-rabies traditional practice in Chilga district, Central Gondar administrative zone. From the total number of respondents, 222 (28.9%) households reported suspected rabies exposure in their family member in the two years study period. In these households, 424 suspected rabies exposure cases were reported during the study period, of which 375 were less risky exposure (if rabid dog died in the immediate neighbor, people feel exposed to rabies irrespective of any contact with rabid dog and take traditional treatment) and 49 were risky exposure with bite (bite with rabid dog resulting bleeding scratch or open wound) of suspected rabid dog (Table 1). Out of the 49 risky exposed cases, 44 (90%) victims visited health centers and 5(10%) visited traditional healers to get traditional diagnosis and treatment for rabies. From those who visited health centers, 32 people received complete dose of PEP, 10 incomplete dose and 2 did not take the PEP. A total of 4 rabies deaths were reported by respondents (768 respondents having average household size of 7 people) which resulting in 37 deaths/100, 000 people per year in the district.

Table 1: The nature of rabies exposure and deaths from 768 households of Chilga district

Rabies related events	2017/18	2018/19	Total
Less risky exposure	142	233	375
Risky exposure	22	27	49
Deaths	3	1	4

In the 49 risky exposures, dog bite sites include: 1 in the head/neck region, 10 on arms/hands, 33 on the legs and 5 on the trunk (Table 2). From the total four deaths, three died following bite of legs, whereas one death was due to hand bite. Three of the dead persons did not receive PEP, while the remaining one started PEP but died before completing the dose. The age of died persons were between 15 and 44 years.

Table 2: Rabies suspected cases (n=49) bite sites (head, hand, trunk, and leg), age category and death status

Age categories	Bite sites of the victims and death				
	**Head/neck**	**Hand**	**Trunk**	**Leg**	**Total**
0-4	0	0	1	1	2
5-14	0	1	2	8	11
15-29	1	6	1	15*	23
30-44	0	3*	1	5**	9
45-59	0	0	0	2	2
60-69	0	0	0	2	2
70-79	0	0	0	0	0
80+	0	0	0	0	0
**Total**	**1**	**10**	**5**	**33**	**49**

*one death

**two death

3.1.1. Health burden estimation in terms of disability adjusted life years (DALYs)

The total DALYs due to YLL was estimated at 1576.5 DALYs/100,000 people per year and the highest YLL was found in the age group between 15-19 in male, whereas between 35-39 in female (Table 3).

Table 3: The estimation of years of life lost (YLLs) due to premature death from 2017/8-2018/9 (N= 5376 people (768 households * 7people))

Age categories	Male			Female			Person
	**No of death**	**Life expectancy**	**YLLs**	**No of death**	**Life expectancy**	**YLLs**	**Total YLLs**
0-4	0	65.7	-	0	68.8	-	-
5-9	0	62.9	-	0	66	-	-
10-14	0	58.7	-	0	61.7	-	-
15-19	1	54.1	54.1	0	57	-	54.1
20-24	0	49.7	-	0	52.5	-	-
25-29	0	45.5	-	0	48	-	-
30-34	1	41.2	41.2	0	43.5	-	41.2
35-39	0	37	-	1	39.2	39.2	39.2
40-44	0	32.9	-	1	35	35	35
45-49	0	28.9	-	0	30.9	-	-
50-54	0	24.9	-	0	26.8	-	-
55-59	0	21.1	-	0	22.8	-	-
60-64	0	17.4	-	0	18.8	-	-
65-69	0	14	-	0	15.1	-	-
70-74	0	10.9	-	0	11.8	-	-
75-79	0	8.3	-	0	8.9	-	-
80-84	0	6.1	-	0	6.5	-	-
85+	0	4.4	-	0	4.8	-	-
**Total**	**2**		**95.3**	**2**		**74.2**	**169.5**

Health burden is expressed in terms of standard disability adjusted life years (DALYs) and normally calculated as in the summation of YLL and YLD. However, in our analysis years of life lived with disability (YLD), which was considered from NTV adverse effect, was zero due to the absence of NTV adverse effects report in the district during the study period and the DALY was the same as YLL which was 1576.5 DALYs/100,000 people per year in district. Higher DALYs was estimated in males (56%) than females (44%) (Table 3).

3.2. Economic Burden of Rabies

3.2.1. Estimated economic loss of rabies in humans

Total costs of seeking PEP per household and per district were 73.7 ETB and 2.4million ETB/year respectively. Direct medical cost (wound care and PEP vaccination) contributed 0.7million ETB of the overall cost and indirect non-medical costs (transportation, accommodation and food costs) contributed 1.7million ETB/year in the district (Table 4).

Table 4: The costs associated with human rabies cases in seeking of PEP from 2017/8 to 2018/9 for 768 households

Years	No of human treated	No of PEPs (doses)	[a]Direct medical cost (birr)	[b]Indirect non-medical cost (birr)	Total cost (birr)
2017/8	17	245	11759	41174	52933
2018/9	25	414	21537	38733	60270
**Total**	**42**	**659**	**33296**	**79907**	**113203**

[a]Average costs of direct medical cost per person =792.8ETB; [b]Average indirect non-medical cost per person=1902.5 ETB.

During the study period, 200 of 768 respondents went to traditional healers. The major components of costs associated with traditional practice identified were traditionally diagnosis (TDx) cost which amount 0.35million ETB/year, traditional treatment (TRx) cost which estimated to 0.52million ETB/year and other costs (travel and food cost) that amount 0.14million ETB/year (Table 5). Total costs associated with anti-rabies traditional practice expressed per household and per district were 31.2 ETB and 1million ETB/year respectively.

Table 5: Cost associated with traditional anti rabies practice from 2017/8 to 2018/9 for 768 households

year	No of TDx	No of TRx	[a]Total TDx cost(ETB)	[b]Total TRx cost(ETB)	[c]Other costs(ETB)	Total cost(ETB)
2017/8	152	133	5550	9010	2967	17527
2018/9	244	222	11100	15790	3557	30447
**Total**	**396**	**355**	**16650**	**24800**	**6524**	**47974**

[a]TDx = based on average traditional diagnosis of cost 42 ETB, [b]TRx = based on average traditional treatment cost 70 ETB per person and [c]Other costs = based on other average cost of 16.5ETB per person.

3.2.2. Estimated economic loss due to occurrence of rabies in livestock

Cattle and donkey were the most frequently bitten animals by a suspected rabid dog. The estimated direct economic losses due to the occurrence of rabies in livestock were, therefore, associated with the number of exposed and died cattle and equine due to rabies. Most of the animals bitten by a suspected rabid dog did not develop rabies. However, in case of cattle, these animals were slaughtered and the meat sold to their neighbors as a precautionary measure, as owners did not want to face the risk that their cattle gradually develop clinical signs.

Because of this cattle bitten by a suspected rabid dog had some salvage value. In contrast, donkeys that were considered to be rabid had no salvage. Thus, all exposed animals ended up with death as a result of the disease or due to the precautionary cull and the cost estimated include both dead and salvaged animals (Table 6). Based on these deaths and cull the total rabies cost in livestock per household and district were 350.9 ETB and 11.4million ETB/year respectively.

Table 6: Costs of rabies in livestock (cattle and donkey) in the study period from768 households

	Cattle			Donkey		
**Year**	**Average cost/head(birr)**	**#Culling**	**Total cost(birr)**	**Average cost/head(birr)**	**#Death**	**Total cost(birr)**
2017/8	7005.5	27	189150	2055.5	9	18500
2018/9	8312.5	36	299250	2469.2	13	32100
**Total**		**63**	**488400**		**22**	**50600**

3.2.3. Estimated costs of dog vaccination

During the study period (from2017/8-2018/9), over 3475 dogs were vaccinated against rabies in the district. The total annual cost of vaccination of dogs expressed per household and district wer

e about 2 ETB and 51,438 ETB/year respectively (Table 7). The cost of the vaccine accounted the largest (~84%) proportion of the total vaccination cost.

Table 7: The costs of prevention of rabies in dog from2017/8-2018/9 in the district (32,496 house holds)

Year	No of dog vaccinated	[a]Dog vaccine cost(birr)	[b]Vaccinator cost(birr)	[c]Dog owner cost(birr)	Total cost(birr)
2017/8	1565	39125	2450	4680	46255
2018/9	1910	47750	3150	5720	56620
**Total**	**3475**	**86875**	**5600**	**10400**	**102875**

Average costs associated with [a]vaccine/dog=25ETB, [b]vaccinator/dog=1.6ETB and [c]dog owner/dog=3ETB

3.2.4. Total annual economic cost due to rabies in the district

The overall economic cost of canine rabies at district level from September 2017 to September 2019 was estimated as 457.4 ETB/household and14.9 million ETB/year in the district. The livestock losses contributed the major share (76.7%) of the total annual cost followed by human PEP associated cost contributed (16%) (Figure 2).

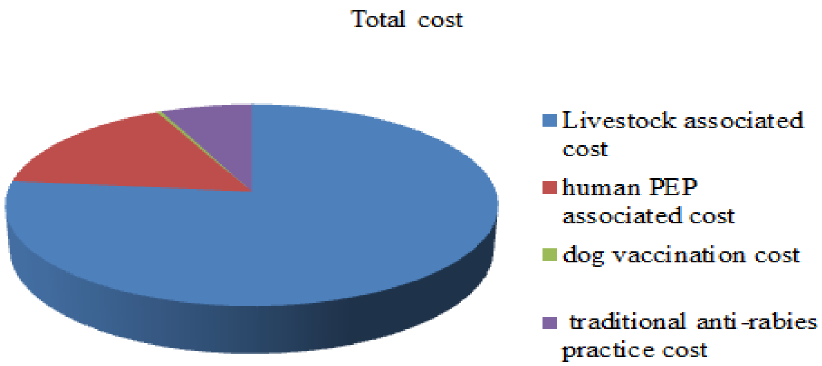

Figure 2: Proportional distribution of total rabies costs

4. DISCUSSION

This study reported a high burden of rabies in which 28.9 % of sample households reported a suspected rabies exposure in their family member with total number of 424 rabies exposures and 37 deaths/100,000 people per year. This reported death is higher than 0.7, 4.2 and 2.9 deaths/100,000 people per year reported respectively in Bishoftu, Lemuna bilbilo and Yabelo districts in other parts of Ethiopia [29]. The current study is also reported higher number of deaths than the previous national estimate in Ethiopia [19], which was 1.6 rabies deaths/100,000 people. The current estimate is also high when compared to estimates from other countries such as the 914 human deaths reported from 2005 to 2014 in Vietnam, where the death rate was 0.11rabies deaths/100,000 population [28]. The community-based interview approach used in this study, despite some potential recall bias, likely captured incidents that may have been overlooked in official reporting systems. This could explain the high number of deaths reported in the current study, but caution is needed as this is a small study that covered small geographical areas and time span.

Among the exposure reports, 88% were less risky exposures, in which the people take unnecessary measures due to lack of good knowledge about the nature of rabies transmission. These were not true exposures, but people were afraid and had taken measures mostly traditional medicines/practices. Creating awareness about risky exposures and non-risky exposures associated with rabies to the community would avoid unnecessary worry and measures during rabies incident.

A significant number of DALYs were lost due to premature human deaths. Different reasons for not visiting health facilities and taking PEP on time were mentioned by respondents for these rabies caused deaths. The family for two death victims did not recognize the biting dog was affected by the disease. Another one victim started PEP late and showed clinical sighs while taking the PEP. The other victims had done nothing due to inaccessibility of PEP vaccine.

The total public health burden in the district due to rabies was estimated to be1576.5 DALYs/100,000 people per year. This is high, when compared to 48.7, 297.5 and 239.8 DALYs/100,000 people per year reported by Beyene *et al.* [29] at Bishoftu, Lemuna-bilbilo and Yabelo districts respectively. When DALYs were compared between male and female, DALYs in males (886.4 DALYs/100,000 people per year) was higher than in females (690.1 DALYs/100,000

people per year). This DALY difference is related to the difference in the age of death (male was died at younger age than female) not to the number of death (equal number of people died in each sex category).

This study had also provided an estimate of the direct and indirect economic costs due to rabies in the district during the study period. The total annual economic cost was estimated to be 457.4 ETB/household and 14.9 million ETB/per district. The largest portion (76.7%) of rabies costs in the district was associated with livestock losses and only small portion (0.3%) of the cost was associated with dog vaccination. The reason for this low cost of dog vaccination is that only few numbers of dogs were vaccinated. This indicated that there were low rabies control and prevention activities in the study area.

The human rabies cost estimated in this study included only expenditure in the form of medical and non-medical expense. The economic impact of human rabies goes beyond expenses by causing productivity loss due to illness and premature death but this was not captured in this study as it involves complex data and analysis which was outside of the study scope.

This study was done by interviewing a sample of households about their experience of rabies related events in their family member and livestock two years preceding the interview. This might introduce recall bias in the data collected but given the dramatic nature of the disease and obvious symptoms and invariably fatal consequence, the recall problem would be in consequential. This has been similarly justified in the published literatures [30, 31].

Generally, the study showed a significant public health and economic burden of the disease in the district that needs a control intervention specially strengthening dog vaccination, which was observed to be very low during the study, could have significant impact in reducing both public health and economic burden. Further study based on follow up of a confirmed outbreak of rabies is needed to refine the estimates of public health and economic burden.

Author contribution statement:

Tewodros Birhan Engida: designed the study, collected and analyzed the data and drafted and revised the paper.

Amsalu Misgie Molla: designed the study, Analyzed and interpreted the data and revised the paper.

Wassie Molla: designed the study, Analyzed and interpreted the data and revised the paper.

Wudu Temesgen Jemberu: conceived the study, designed the study, Analyzed and interpreted the data and revised the paper. All authors contributed to the article and approved the submitted version.

ACKNOWLEDGMENTS

The authors would like to thank the house holds who participated in the study and for providing information needed for the study and the University of Gondar was highly acknowledged for providing the facilities needed to accomplish this study.

Conflict of interest

The authors declare no conflict of interest

6. REFERENCE

[1] King AA, Turner GS. Rabies: a review. *Journal of Comparative Pathology*. 1993 Jan 1;108(1):1-39.

[2] Wandeler AI, Bingham J. Dogs and Rabies. In: Macpherson CN, Meslin FX, editors. Dogs, zoonoses, and public health. Wallingford: CABI Publishing, 2000. Pp. 436-9.

[3] World Health Organization. Oral immunization of dogs against rabies: report of the sixth WHO consultation, Geneva, 24-25 July 1995. InOral immunization of dogs against rabies: report of the sixth WHO consultation, Geneva, 24-25 July 1995 1998.

[4]Randall DA, Marino J, Haydon DT, Sillero Zubiri C, Knobel DL, Tallents LA, et al. An integrated disease management strategy for the control of rabies in Ethiopian wolves. *Biological Conservation*. 2006 Aug 1;131(2):151-62.

[5] Green AL, Carpenter LR, Dunn JR. Rabies Epidemiology, Risk Assessment, and Pre and Post-Exposure Vaccination. *Veterinary Clinics: Exotic Animal Practice.* 2011 Sep 1; 14(3):507-18.

[6] Jackson AC, Warrell MJ, Rupprecht CE. Rabies in Human In. Elsevier/Academic Rabies.2nd ed. Amsterdam. The Netherlands; 2007.

[7] Shankar VI, Dietzschold BE, Koprowski HI. Direct entry of rabies virus into the central nervous system without prior local replication. *Journal of virology*. 1991 May; 65(5):2736-8.

[8].Wunner WH. Rabies in the Americas. *Virus Res.* 2005; 111(1):1-4. doi: 10.1016/j.virusres.2005.03.005

[9] Colville JL, Berryhill DL. Handbook of zoonoses, identification and prevention Mosby Elsevier, St. Louis (MO). 2007.

[10] Briggs DJ. Rabies vaccines. In: Jackson AC, editor. Rabies. Amsterdam: Elsevier Academic Press; 2007. p. 505–16. DOI: 10.1016/B978-012369366-2/50015-4

[11]Hampson K, Coudeville L, Lembo T, Sambo M, Kieffer A, Attlan M, et al. The Global Alliance for Rabies Control Partners for Rabies Prevention. Estimating the Global Burden of Endemic Canine Rabies. *PLoS Neglected Tropical Diseases*. 2015; 9: e0003709. http://doi.org/10.1371/*journal.pntd*.0003709.

[12] Knobel DL, Cleaveland S, Coleman PG, Fèvre EM, Meltzer MI, Miranda ME, *et al*. Re-evaluating the burden of rabies in Africa and Asia. Bulletin of the World Health Organization. 2005; 83(5): 360-368. PMID: 15976877; PMCID: PMC2626230.
[13] Meslin FX, Briggs DJ. Eliminating canine rabies, the principal source of human infection: what will it take? *Antiviral research.* 2013 May 1; 98(2):291-6.
[14] Blanton JD, Palmer D, Rupprecht CE. Rabies surveillance in the United States during 2009. *Journal of the American Veterinary Medical Association*. 2010 Sep 15; 237(6):646-57.
[15] Rupprecht CE, Briggs D, Brown CM, Franka R, Katz SL, Kerr HD, et al. Use of a reduced (4-dose) vaccine schedule for post exposure prophylaxis to prevent human rabies: recommendations of the advisory committee on immunization practices. *MMWR Recomm Rep. 2010 Mar 19; 59(RR-2):1-9.*
[16] Undurraga EA, Meltzer MI, Tran CH, Atkins CY, Etheart MD, Millien MF *et al*. Cost–effectiveness evaluation of a novel integrated bite case management program for the control of human rabies, Haiti 2014–2015. *Am J Trop Med Hyg*. 2017; 96(6):1307–17.
[17]Ali A, Mengistu F, Hussen K, Getahun G, Deressa A, Yimer E. et al.Overview ofRabies in a nd around Addis Ababa, in Animals Examined in EHNRI Zoonosis Laboratory Between, 2003 and 2009. *Ethiopian Veterinary Journal*. 2010; 14: 91-101.
[18] Yimer E, Newayeselassie B, Teferra G, Mekonnen Y, Bogale Y, Zewde B, et al. Situation of rabies in Ethiopia: a retrospective study 1990-2000. *Ethiopian Journal of Health Development*. 2002; 16(1):105-12.
[19] Deressa A, Sefir D, Mamo H, Ali A, Akililu M, Getahun G. Rabies diagnosis and surveillance for the period 2010–2012 in Ethiopia. In11th SEARG meeting, Dar es Salaam; 2013. pp11-15.
[20] Moges N. Rabies in Ethiopia: Review Article. *Academic Journal of Animal Diseases*. 2015; 4: 74-81.
[21] Tenzin T, Dhand NK, Gyeltshen T, Firestone S, Zangmo C, Dema C, et al. Dog bites in humans and estimating human rabies mortality in rabies endemic areas of Bhutan. *Plos Neglected Tropical Diseases*. 2011; 5 (**11**): e1391. https://doi.org/10.1371/*journal.pntd*.000139.

[22] Central Statistical Agency (CSA). Federal Democratic Republic of Ethiopia Central Statistical Agency. Population and Housing Census Commission: Summary and Statistica l Report of the 2007; Addis Ababa, Ethiopia.

[23]Chilga livestock office. Chilga livestock development and agency office annual unpublished numbers; 2019.

[24] Chilga agricultural office. Chilga district socio-economic unpublished documents; 2019).

[25] Kotrlik JW, Higgins CC. Organizational research: Determining appropriate sample size in survey research appropriate sample size in survey research. Information technology, learning, and performance journal. 2001 Jul 12; 19(1):43.

[26] Dohoo IR, Leslie K, DesCôteaux L, Fredeen A, Dowling P, Preston A, et al. A meta-analysis review of the effects of recombinant bovine somatotropin: 1. Methodology and effects on production and nutrition related parameters. *Canadian Journal of Veterinary Research.* 2003 Oct; 67(4):241.

[27] Üstün TB, Chatterji S, Mechbal A, Murray CJ, WHS Collaborating Group. The world health surveys. Health systems performance assessment: debates, methods and empiricism. Geneva, World Health Organization. 2003; 797.

[28] Shwiff SA, Brown VR, Dao TT, Elser J, Trung HX, Tien NN, et al. Estimating the economic impact of canine rabies to Viet Nam 2005–2014. *PLOS Neglected Tropical Diseases*. 2018 Oct 11; 12(10):e0006866. https://doi.org/10.1371/*journal.pnt*d.0006866.

[29] Beyene TJ, Mourits MC, Kidane AH, Hogeveen H. Estimating the burden of rabies in Ethiopia by tracing dog bite victims. PLoS one. 2018 Feb 21; 13(2):e0192313.

[30]Jost CC, Mariner JC, Roeder PL, Sawitri E, Macgregor Skinner GJ. Participatory epidemiology in disease surveillance and research. *OIE Revue Sci.et Tech*. 2007; 26 (3): 537–549.

[31] Rufael T, Catley A, Bogale A, Sahle M, Shiferaw Y. Foot and mouth disease in the Borana pastoral system, southern Ethiopia and implications for livelihoods and international trade. Tropical animal health and production. 2008 Jan; 40(1):29-38.

7. APPENDICES

Appendix 1: Questionnaire to be administered to households about to rabies victims/families

Questions to rabies victims/their families exposed between September 2017 and September 2019

Respondent to participate: Agree ---------Disagree--------- *Date of interview* ---------------------

Name of respondent (household head) --------------------------------------district-------------------

kebele--------------Sex-----------age---------address/phone No--------------------------------

Human (Part one)

1. Do you know, what rabid dog/animal means? A. Yes B. No

If yes, what are typical symptoms: 1. A dog disease with aggressiveness and salivation

2. A dog disease with paralysis and salivation

3. A dog disease with restlessness and salivation

2. Were/was there any family member bitten/suspected to be bitten by rabid dog since September 2017? A. Yes B. No

For each bitten or suspected of bitten (exposed) family member complete the following table

No.	Status of exposure (as non bleeding, bleeding scratch, open wound)	Status of the dog(confirmed, only clinically suspected, later found non rabid	Date of exposure (at least month)	Age of the victim	Sex of the victim	Site of exposure such as (Head…)	Measure taken during exposure (wound management and or PEP and or traditional treatment)	Fate of the victim

For each exposed family number complete the following cost related table

Victim no	Convention treatment						Traditional treatment			
	Wound care cost if any	No of PEP doses if any	Hospital cost (drug and bed) cost of any	No of days of illn ess	The expen se of the a ccompany during period of illness	Transport cost of the victim and his accompan y	Diagn osis, Treat ment cost	Tran sport cost	Days of off home treat ment	Expense of the accompan y

Livestock (part two)

3. Were/was there any rabid/rabies suspected cases among your animal since September 2017?

A. Yes B. No

If yes, complete the following table

No	Date of exposur e(atleas t month)	Species of the animal	Age of ani mal	Sex of anim al	Status of the anim al(rabid or suspe cted)	The nature of exposur e (minor, Moderate, severe)	If treated conventio nally cost of treatm ent	If treated traditiona lly cost o f treatme nt	No of day s spent for get the affected animal treated	If died the estima ted price	If Salva ged the estimate value lost

Questionnaire to be administered to households about traditional practice

1. Do you know traditional healers who give anti-rabies traditional medicine?

A. Yes B. No

2. Mostly, what do you and your community do when humans/animals are exposed to rabies?

A. traditional treatment B. modern treatment in health centers

2.1. If traditional does it include diagnosis or only treatment?

A. only treatment B. both treatment and diagnosis

2.2. If traditional, what type of treatment is used in the traditional method?

A. spiritual B. non spiritual C. mixed

2.3 If the treatment is non spiritual, what is the medicine made of:

A. herbs B. animal products C. others

3. Have you/families diagnosed/treated when suspected/bitten by rabid dog?

A.Yes B. No

3.1. If yes, do you have experience of misdiagnosis in the traditional treatment?

A. Yes B. No

3.2. If yes, do you have experience of treatment failure in the traditional treatment?

A. Yes B. No

If yes, what do you think is the failure?

3.3. Do you have experience of success in traditional treatment? A. Yes B. No

4. Do you have experience of failure after modern treatment of human rabies exposure cases?

A. Yes B. No

If you yes, what do you think will be the reason---

----

5. Do you have experience of failure after modern treatment of animal rabies exposure cases?

B. Yes B. No

If you yes, what do you think will be the reason---

----

6. Do you have experience of success after modern treatment human exposure cases?

A. yes B. No

7. Do you have experience of success after modern treatment animal exposure cases?

A. Yes B. No

8. Do you think that traditional healers can cure human bitten by a rabid animal?

A. Yes B. No

9. Do you think that traditional healers can cure an animal bitten by a rabid animal?

A. Yes B. No

10. Do you think that traditional healers can cure human once started to show clinical signs of the rabies? A. Yes B. No

11. Do you think that traditional healers can cure an animal once started to show clinical signs of the rabies? A. Yes B. No

12. If using traditional treatment in humans does it have side effect in humans? A. Yes B. No

If yes what ?---
----

13. If using traditional treatment in animals does it have side effect? A. Yes B. No

14. Have ever used preventive traditional medicine for rabies i.e. medicine taken before any suspected exposure of rabies? A. Yes B. No

15. If yes do you think it is effective. A. Yes B. No

In traditional treatment how do traditional healers determine the dose? -------------------------------
----

16. How medicine administration performed? A. by healers B. by patients/them selves

17. Would they use anti-dotes? A. Yes B. No

18. If yes, what are? ---

Part three

Dog (Questions for livestock office from September 2017 to September 2019)

1. How many dogs have vaccinated in the district from September 2017 to September 2019?

2. How much birr have expensed for vaccinators-------------------transportation of vaccine---------------------------------in district?

3. The estimated costs of owners for vaccinating their dog? --------------------------------

4. Full dose of canine rabies vaccine cost? --

Click here to access/download
Supporting Information
S1 Data. Raw data and summaries.xlsx

Click here to access/download
Supporting Information
S2. Questionnaire.docx

Click here to access/download
Supporting Information
S3. Ethical Review Statment.pdf
