# Supplementary material for: Health and economic burden of canine rabies in Chilga district, centeral Gondar Zone, Amhara Region, Ethiopia
Source: PLoS Negl Trop Dis. 2026 Apr 20;20(4):e0014196. doi: 10.1371/journal.pntd.0014196 (PMC13124050; doi:10.1371/journal.pntd.0014196)
Supplement: Attachment — Submitted filename: Response to Reviewers.docx [file pntd.0014196.s004.docx]

**Editor and Reviewers comments:**

**Reviewer #1: Introduction**

**Comment:** Line 63: Rabies is caused by any member of the Lyssavirus genus, not just 'a' member. This sentence can be rewritten as "Rabies is a neglected tropical disease caused by members of the Lyssavirus genus in the Rhabdoviridae family"

**Response:** Accepted and revised accordingly (Line 63).

**Reviewer #1:** **Methodology**

**Comment 1:** How were households within Kebeles selected? The manuscript states 'systematically' but this does not provide enough detail either for replication or to judge potential for selection bias. The method of household selection within Kebeles must be more fully described.

**Response:** More descriptions about the selection of kebeles and households are provided in Lines 151-62.

**Comment 2:** What proportion of respondents were excluded because of lack of familiarity with rabies? If substantial, this could underestimate the burden of rabies cases. The number and proportion of respondents excluded for this or other reasons should be reported in the results, perhaps in the form of a participant flow diagram if the number and reasons for exclusion warrant.

**Response:** We thank the reviewer for this valuable comment. By chance all respondents were familiar with rabies; therefore, no participants were excluded from the study due to lack of familiarity. We included this information in the results in this revision (Line 308-310)

**Comment 3:** There seem to be two slightly different versions of the questionnaire: one included as Appendix 1 and another as S2 supplemental file. The Appendix 1 seems to map most directly onto the raw data in S1 and so I assume this is the correct version.

**Response:** We thank the reviewer for carefully noting this issue. Appendix 1 (now supplementary material 1) is the correct and final version of the questionnaire and corresponds directly to the raw data provided in Supplementary File S1 (now S2). The version included as Supplementary File S2 was an earlier draft included inadvertently. This has now been removed.

**Comment 4:** The authors seem to be using a de facto case definition of canine rabies based on typical symptoms as 1. A dog disease with aggressiveness and salivation, 2. A dog disease with paralysis and salivation, or 3. A dog disease with restlessness and salivation. If so, this could be summarized and stated in the methods: We defined a clinically suspected case as a dog with hypersalivation plus aggressiveness and/or restlessness and/or paralysis. The authors should note that this differs from the WHO definition of a suspect cases (being an animal that presents with any of the following clinical signs: Hypersalivation; Paralysis; Lethargy; Unprovoked or abnormal aggression (biting two or more people or animals, and/or inanimate objects); Abnormal vocalization; Diurnal activity of nocturnal species) but that their definition may provide a better working definition for household reporting (on the flip side, their definition would miss cases that do not show hypersalivation, or that show lethargy or abnormal vocalization in addition to hypersalivation without restlessness, aggression or paralysis). The authors must present their case definition clearly and discuss its pros and cons compared to the standard WHO case definition.

**Response:** We thank the reviewer for this insightful comment, we accepted the comment and revised accordingly. We acknowledge that this operational definition differs from the broader WHO case definition, While our approach may enhance specificity for recognition by household reporters in the study setting, we recognize that it may underestimate cases that do not exhibit hypersalivation or that present only with signs such as lethargy or abnormal vocalization. We described the implication of this limitation discussion in this revision (Line 490-494).

**Comment 5:** The authors do not define 'risky' vs. 'less risky' exposure. Here, they should follow the WHO classification of exposures as Category I (Touching or feeding animals, licks on intact skin (no exposure)), Category II (Nibbling of uncovered skin, Minor scratches or abrasions without bleeding (exposure)), or Category III (Single or multiple transdermal bites or scratches, contamination of mucous membrane or broken skin with saliva from animal licks, exposures due to direct contact with bats (severe exposure)). The authors’ category of 'less risky exposure' seems to include no contact with the dog - this could be a 'Category 0'.

**Response:** Thank you for this critical observation and for the constructive suggestion. As suggested, we revised the methodology to align our exposure categorization with the WHO, 2018 classification (Line 190-198). Our Less Risky Exposure category was defined to include “Category 0” (perceived exposure without physical contact) and “Category I “(touching or feeding animals, licks on intact skin); and Risky Exposure was defined to include “Category II” (nibbling of uncovered skin, minor scratches or abrasions without bleeding) and “Category III” (single or multiple transdermal bites or scratches, contamination of mucous membranes or broken skin with saliva).

**Comment 6:** The authors should report the annual incidence of 'risky exposures' to suspect rabid dogs per 100,000 people in the Results and compare it to similar estimates from other studies in the Discussion (ideally in the context of a standard definition of 'risky exposure' as WHO Category II/III or Category I/II/III, depending on what underlying criteria were used).

**Response:** Exposure per 100,000 per year is calculated and included in the results section in this revision (Lines 315-317 and Table 1).

**Comment 7:** I'm confused about the animal exposures in the livestock economic analysis. In the Excel sheet of the raw data, all 85 exposed animals (86 is we count the one caprine) are classified as 'rabid' in the status variable (in the questionnaire, the options seems to be 'rabid' or 'suspect'). Does this mean they were laboratory confirmed? But if the 63 exposed bovines were culled after exposure, how could they have been confirmed? Did all the equines go on to develop clinical signs of rabies? Were they confirmed or suspect? It is very unlikely that all exposed animals would develop disease - in fact, the authors say in lines 299-300 that 'Most of the animals bitten by a suspect rabid dog did not develop rabies", yet in lines 304-305 they say "Thus, all exposed animals ended up with death as a result of the disease..." (Referring to donkeys, which were not culled after exposure because of the lack of salvage value). The authors need to clarify this section.

**Response:** Thank you for spotting these confusing texts. The caprine was excluded from the analysis as there was data only in one goat. The rabies status question answered rabid for all exposed livestock in the raw data was an error (corrected and uploaded in the Supplementary Material 2). All cattle were culled as precautionary measure and all exposed donkeys were claimed to rabid and died. The description of the rabies costs in livestock in section has been revised and clarified (Line 393-406, Table 6).

**Reviewer #2: Methods**

**Comment #**1. sampling: The paper needs to better explain the sampling stages. Although the authors doubled the sample size for clustering effect, they have not commented on the homogeneity or heterogeneity of the kebeles. Additionally, they did not estimate the sample using previous estimates and should provide a rationale for this. How the households who refused the survey, were handled needs to be explained. Also, how did they arrive at 25 sample number for kebeles ? Did they use population proportionate to size or something? There is also a need for an explanation of how households within each kebeles were selected? Did they have a list of all households and use random sampling, or follow systematic sampling (every 10th household), or some GIS-based method? Furthermore, were the 768 households evenly divided among the 25 kebeles? The paper would be strengthened if these questions are addressed in the methods section.

**Response:** Thank you for these comments. The sampling methods and sample size description has been thoroughly revised to address the valid comments in this revision (section 2.2.1)

**Comment #2:** Extrapolation of sample findings and uncertainty of estimates: The methods section should explain how the findings from the sample were extrapolated to the district or population level. The tables should also describe the extrapolation results. The assumptions and formulas used for extrapolation should be clearly stated in the methods section. Also, there are uncertainties in the findings (95% CI) and the cost (95% CI). These should be stated, and the extrapolated estimates of cost should also reflect these uncertainties and include confidence intervals. The sources of data for costs, such as average medical costs, vaccine costs, etc., and other parameters used should be listed (preferably in a separate table).

**Response:** The methods of confidence interval estimation and extrapolation to the district population has been included the material and methods section 2.5 (line 263-280) and 95% confidence intervals as indicator of uncertainty for the estimate are included in the results (Table 1, 4,5; lines, 325,358,375, 378,383,403,427 )

**Comment #3:** Inclusion of Dog Rabies Vaccination Costs: Vaccinating dogs against rabies prevents rabies in humans, and those costs should not be included in the total costs of rabies. Cost-of-illness studies generally include direct and indirect medical costs incurred once a disease or illness affects a population. They do not account for the costs associated with preventing the disease. Therefore, BCG vaccine costs are not included in the cost of TB, or COVID vaccine costs are not included in the cost of COVID, or seat belt costs are included in the cost of accidental injuries or deaths. The rabies cases occurred despite 3475 dogs being vaccinated. Dog vaccination costs are relevant when designing rabies elimination programs or prevention. Hence, it cannot be included in the costs of rabies. Though Shwiff et al (ref no 28) have included it in their paper, I strongly recommend not including it in this paper. If required, the dog vaccination costs can be presented separately.

**Response:** The dog vaccination cost parts has been removed from the manuscript as suggested

**Comment:** Mention Ethiopia’s burden here?

**Response:** We thank the reviewer for this valuable comment. In response, we have revised the manuscript accordingly and incorporated the suggested points to better highlight Ethiopia’s rabies burden (Line 83-84).

**Comment:** Please quantify the assertion with numbers

**Response:** we have revised the manuscript to address this comment (Line 100-105).

**Comment:** Give deeper reasons for this. Is rabies a notifiable disease in Ethiopia? Why is rabies deaths not recorded properly? How is dog population estimated etc.?

**Response:** We thank the reviewer for this important comment and we have revised the manuscript accordingly (Line 110-120).

**Comment:** mention the data collection period?

**Response:** Accepted and revised accordingly (Line 145).

**Comment:** Describe exactly how many stages were there. How sampling was done at each stage? How did you handle homogeneity or heterogeneity between clusters?

**Response:** Thank you for the comment. We have revised the sampling method (section 2.2.1).

**Comment:** why was the estimate of rabies 12 exposure cases or 1.6 rabies deaths per 100000 not used to calculate sample size?

**Response:** The figures 12 human exposure cases per 100, 000 population and 1.6 rabies deaths per 100, 000 population come from official national surveillance estimates which were based on passive surveillance, likely underreported , and not specific to the study’s target population. We preferred assuming the 50% to insure sufficient households are used for study instead of using those passive surveillance estimates which are usually underreported.

**Comment:** Are all the kebeles homogeneous?

**Response:** The kebeles in our study district are unlikely to be completely homogeneous in terms of population, dog ownership, or rabies exposure risk. Half of the kebeles were included in the sample and this is believed to represent the district. As the households may tend to be similar within kebeles which may introduce clustering, the sample size taken assuming simple random sampling was doubled to account clustering within kebeles. Descriptions about these have been added in this revision (Line 154-156; 174-176).

**Comment:** how were non respondents for e.g. those who refused to give consent or were absent handled? How many non-respondents and how were the households replaced.

**Response:** Thank you for the comment. By chance all respondents were participated in the study.

**Comment:** mention the source of the life table used for this

**Response:** Reference provided (Line 212)

**Comment:** mention the disability weight used and source

**Response:** Disability weight and reference provided (Line 219)19)9)

**Comment:** Line 188: should be cost of rabies vaccination in dogs

**Response:** This part removed as suggested

**Comment:** Line 190: Major concern in including these costs. The costs can be presented separately.

**Response:** This part removed as suggested

**Comment:** Line 202: mention the exact prices used for cattle and quinine and their source

**Response:** The estimated prices of culled cattle and died donkey were asked from the respondents and the prices vary from animal to animal (the priced for each animals can be found in the raw data in Supplementary Material 2). These prices were used to estimate average losses per animal and per household as indicated in Table 6.

**Comment:** Line 214: Please explain clearly how the findings from sample were extrapolated to the population level. Provide formulas and assumptions**.**

**Response:** The material and method section 2.5 has been revised to indicate how confidence interval estimation is done to extrapolate the findings from sampled households to the district population of households (Line 266-274).

**Comment:** Line 216: mention source. Also where did the sep. 2017 to sep. 2019 numbers adjusted for inflation? Which year USD is being presented 2025 USD or 2019 USD?

**Response**: The exchange rate was provided to help the international readers to easily convert costs to USD. Otherwise, all costs were collected and reported in ETB as they occurred over the two study years. No inflation adjustment was made from Sept 2017-2019. The reported costs are average ETB value of this period. This is mentioned in this revision (Line 282-83).

**Comment:** Line219-226: can be condensed to two sentences

**Response:** Accepted and revised accordingly (Line 286).

**Comment:** Line 238-239: Exposure classification into less risky and risky needs to be defined in methods section.

**Response:** Accepted and revised accordingly (Line 190-198).

**Comment:** Line 248: can the table be modified to add columns for those who went to traditional healers and those that came for PEP

**Response:** Information on the number of people sought PEP and traditional treatment are documented in Table 4 and 5.

**Comment:** Line 268: NTV needs to be expanded and explained in methods

**Response:** The comment accepted (line 345) and the disability associated with NTV has been described in the methods section (Line 215-220).

**Comment:** Line 275: The table does not show the calculations for per household and per district. It would be betters if there was confidence intervals around the estimates.

**Response:** This part including the table has been revised to make the calculation clearer and confidence intervals has been included (Line 352-356 and Table 4)

**Comment:** Line 281-282: what is the source for this data and cant the SE be used to get confidence intervals for the estimates

**Response:** As mentioned in the preceding response, this part has been revised to address including this comment.

**Comment:** Line 283-287: The table does not explain how these number were derived from the sample calculations. Please modify the table to reflect these estimates

**Response:** This part including the table has been revised to address the comments (Line 369-375 and Table 5)

**Comment:** Line 293-294: Mention source and also use SE for confidence intervals

**Response:** As in the preceding response

**Comment:** Line 308: mention source and also use SD/SE to get uncertainty about the estimates.

**Response:** The table modified and uncertainty (confidence intervals) included (401-403, Table 6)

**Comment:** Line 309: this can be presented separately and not included in the cost of rabies disease

**Response:** This part about dog vaccination cost has been removed as suggested

**Comment:** Line 310: How many dogs were there in total? What percentage of dogs were vaccinated? How does it compare with national estimates? Dog vaccination prevents rabies in humans. So it cannot be included in cost of rabies diseases. The rabies disease occurred inspite of dog vaccination hence it shows more dogs need to be vaccinated to save "rabies disease costs"

**Response:** As the preceding response

**Comment:** Line 317: Mention source and include confidence intervals

**Response:** As the preceding response

**Comment:** Line 322-323: Make a table of the different cost components or modify the figure to reflect both the cost and percentage

**Response:** The texts and the figure revised as suggested (Line 425-429 and Figure 2).

**Comment:** Line 322-323: Estimated exposures and deaths

**Response:** Accepted and revised accordingly

**Comment:** Line 334-335: discuss the drawbacks in these estimates

**Response:** We appreciate the comment, but the potential drawbacks of these estimate was stated in Line 490-494.

**Comment:** Line 348: Make it “Almost all DALYS”

**Response:** Accepted and revised accordingly (Line 459).

Comment: Line 356-357: discuss why their estiamtes were low and how you study got higher estiamtes ...is it methodiogy is it deaths etc

**Response:** Thank you for the comment, however the justification for the raised comment was sated well in Line 449-453.
